# Impact of the COVID-19 pandemic on breast cancer care in Romania: a cohort analysis from the Oncological Institute of Bucharest

**DOI:** 10.25122/jml-2026-0078

**Published:** 2026-08

**Authors:** Iulia Bistriceanu, Niculae Iordache, Razvan-Andrei Stoica, Carmen Giuglea, Valentin Titus Grigorean, Ileana-Adela Vacaroiu, Mara Mardare, Bogdan Tanase, Octav Ginghina

**Affiliations:** 1 Carol Davila University of Medicine and Pharmacy, Bucharest, Romania; 2 General Surgery Department, Sf. Ioan Clinical Emergency Hospital, Bucharest, Romania; 3 The Academy of Romanian Scientists, Bucharest, Romania; 4 Plastic Surgery Department, Sf. Ioan Clinical Emergency Hospital, Bucharest, Romania; 5 General Surgery Department, Bagdasar-Arseni Clinical Emergency Hospital, Bucharest, Romania; 6 Nephrology Department, Sf. Ioan Clinical Emergency Hospital, Bucharest, Romania; 7 General Surgery Department, Prof. Dr. Alexandru Trestioreanu Oncological Institute, Bucharest, Romania; 8 Pathology Department, Sf. Ioan Clinical Emergency Hospital, Bucharest, Romania

**Keywords:** Romania, breast cancer, COVID-19, time-to-treatment, healthcare disruption, ABS, Association of Breast Surgery, CAR, Canadian Association of Radiology, COVID-19, coronavirus disease 2019, CPBCC, COVID-19 Pandemic Breast Cancer Consortium, CSBI, Canadian Society of Breast Imaging, ESMO, European Society for Medical Oncology, GP, general practitioner, HER2, human epidermal growth factor receptor 2, IOB, Oncological Institute of Bucharest, IQR, interquartile range, MRI, magnetic resonance imaging, NAT, neoadjuvant therapy, NICE, National Institute for Health and Care Excellence, RL, referral letter, SPSS, Statistical Package for the Social Sciences, UK, United Kingdom, US, United States, WHO, World Health Organization

## Abstract

The coronavirus disease 2019 (COVID-19) pandemic profoundly disrupted healthcare systems worldwide, raising concerns regarding delays in cancer diagnosis and treatment. How these disruptions affected breast cancer care during and after the pandemic remains incompletely understood, particularly in Eastern European oncology centers. We conducted a retrospective cohort study of newly diagnosed breast cancer cases treated at the Oncological Institute of Bucharest (IOB), comparing a pandemic cohort (*n* = 73) with a post-pandemic cohort (*n* = 99) with respect to age at diagnosis, tumor laterality, location, stage, grade, receptor subtype, human epidermal growth factor receptor 2 (HER2) status, and intervals across the diagnostic and treatment pathways. We measured the intervals between: General Practitioner (GP) referral and the patient's first presentation to the surgery unit; imaging diagnosis (ultrasound, mammogram, or MRI) and the date of biopsy or surgery; biopsy result and initiation of neoadjuvant treatment; the end of neoadjuvant treatment and surgery; surgery and adjuvant treatment; and consecutive chemotherapy sessions. Between the two cohorts, the median age was similar (56 [47–67] vs. 59 [50–68] years). Similarities were also found in tumor laterality and quadrant location. In the post-pandemic cohort, 31.3% of patients were diagnosed with advanced-stage disease, compared with 20.5% in the pandemic cohort, although this difference did not reach statistical significance. Our study showed that chemotherapy delivery was more efficient post-pandemic, with shorter intervals between treatment sessions (18 [17–19] vs. 21 [21–22] days). Referral-to-presentation times were also shorter post-pandemic (3 [1–8] vs. 6 [2–10] days). In contrast, during the post-pandemic year, the interval between the end of neoadjuvant therapy and surgery remained prolonged. During the COVID-19 pandemic, breast cancer care at a major Romanian oncology center was preserved through adapted clinical pathways. We also found that delays in surgical treatment occurred in the post-pandemic period, and a higher percentage of advanced-stage cases were diagnosed. Our findings suggest that pre-hospital delays and residual system constraints, rather than deficiencies in oncology care, contributed to stage migration in the post-pandemic group.

## Introduction

The most frequently diagnosed malignancy in women remains breast cancer, worldwide, and represents a major contributor to global cancer-related mortality[1]. The incidence had continued to rise in all countries over recent decades, regardless of income level (low, middle, or high). This reflects an improved detection rate, the significant role of lifestyle-associated risk factors, and broader demographic changes. Due to improved screening programs, precise diagnosis with molecular stratification, and extensive treatment strategies, survival rates have improved in many regions [2], but global health inequalities persist. Early detection of new cancer cases through structured screening programs is common in high-income countries, whereas in low-income countries, access to timely diagnosis and comprehensive treatment is limited [3].

In March 2020, the World Health Organization (WHO) declared COVID-19 a global health emergency, triggering a reorganization of healthcare systems worldwide. Pandemic management reshaped hospitals, diverted personnel, postponed elective procedures, and completely stopped screening [4]. As a result, breast cancer care (screening, diagnostic imaging, follow-up consultations, and elective procedures) was significantly impacted.

Professional organizations released clinical guidelines during the COVID-19 pandemic to maintain and support the safe management of breast cancer patients. The European Society for Medical Oncology (ESMO), the Association of Breast Surgery (ABS), the National Institute for Health and Care Excellence (NICE), the Canadian Society of Breast Imaging (CSBI), and the Canadian Association of Radiology (CAR) developed recommendations that helped clinicians to diagnose and treat breast cancer during the pandemic [5].

In the US, the COVID-19 Pandemic Breast Cancer Consortium (CPBCC) was formed and released a decision-making guide for crisis circumstances. The consortium pointed out that the temporary deferral of selected procedures will not influence the long-term outcome of breast cancer patients. It showed that, when possible, reducing patient contact with healthcare facilities was crucial for protecting immunosuppressed individuals and adapting treatment [6].

Telemedicine helped triage patients, provided follow-up consultations, assisted in interpreting laboratory and imaging results, reduced hospital visits, and provided clinical oversight.

Screening programs suffered the most because of these significant changes.

Between March 2020 and June 2020, organized breast cancer screening programs were suspended in many regions. Studies from Italy and England showed a decrease in early-stage diagnoses, an increase in more advanced cases, and, across all patients, an increase in deaths due to these disruptions in screening [7–9]. Currently, neoadjuvant therapy (NAT) for breast cancer involves multidisciplinary collaboration between surgeons, oncologists, radiologists, and others to optimize breast cancer care. NAT, particularly for larger tumors or nodal positivity, can facilitate downstaging and less extensive surgery, potentially improving outcomes. During the COVID-19 pandemic, treatment guidelines were adapted to available resources, and it was shown that neoadjuvant endocrine therapy has proven to be a safe, effective option for postmenopausal women more than for premenopausal women when surgery was delayed [6]. Also, there is evidence that indicates that the outcome is not compromised when postponing surgery up to 8 weeks after neoadjuvant chemotherapy completion [10].

The Romanian National Institute of Public Health issued in 2021 a report about the National Health Assessment and Promotion Program and Health Education that indicates an increase in breast cancer incidence among women from 2011 to 2019 [11]. However, in 2020, newly diagnosed cases showed a decline, with a detection rate of 54.1 per 100 000 women, compared to 62.5 per 100 000 women in 2019 and 67.1 per 100 000 women in 2018 [11]. The report acknowledged that the COVID-19 pandemic influenced the rates, but it does not analyze the mechanisms that drove this trend.

Our study aimed to evaluate the impact of the COVID-19 pandemic and the post-pandemic period on breast cancer management at the Oncological Institute of Bucharest.

The objectives were: (1) to compare the clinicopathological characteristics of newly diagnosed breast cancer patients between the two cohorts (pandemic and post-pandemic), including age at diagnosis, tumor laterality, tumor location, stage, grade, receptor subtype and human epidermal growth factor receptor 2 (HER2) status; (2) to assess differences in time intervals across the diagnostic and treatment pathway; and (3) to determine whether healthcare reorganization during and after the pandemic was associated with delays in care delivery and potential changes in disease presentation.

## Material and Methods

### Study design

This retrospective cohort study was conducted at IOB to assess the impact of the pandemic on the diagnosis and treatment timelines of newly diagnosed breast cancer cases. The study was comparative: patients were divided into two distinct cohorts. The pandemic cohort analyzed data from patients admitted between March 1, 2020, and March 1, 2021. The post-pandemic cohort analyzed data from patients admitted between March 1, 2022, and March 1, 2023. To ensure a clear contrast between the two cohorts, we excluded the transitional year (March 2021 – March 2022).

### Participants

During the study period (March 1, 2020 – March 1, 2021 and March 1, 2022 – March 1, 2023), we identified 641 breast cancer patients hospitalized in oncology or surgical units; however, not all met the inclusion criteria.

The inclusion criteria were: female patients over 18 years of age, a new diagnosis of breast cancer confirmed histopathologically during the study period (March 2020 to March 2021 and March 2022 to March 2023), and eligibility for surgical treatment. The exclusion criteria were: patients under 18 years of age; male patients; recurrent cancer; and female patients with a new diagnosis of breast cancer either outside the study period, or within the study period but not eligible for surgical treatment.

### Data collection

We reviewed medical records to collect data for each eligible patient, including: date of imaging diagnosis (defined as the first radiological investigation suggestive of malignancy, BI-RADS 4 or 5, performed at IOB or externally); type of imaging performed (mammogram, ultrasound, or MRI); date of biopsy; systemic treatment (endocrine therapy and chemotherapy); tumor stage and grade; receptor subtype; HER2 status; date of surgical intervention; and adjuvant treatment (chemotherapy and radiotherapy).

For each patient, we calculated the time intervals, expressed in days, along the diagnostic and treatment pathway. We assessed the referral delay, defined as the interval between issuance of the referral note (by the family doctor) and the patient’s first presentation at IOB; the diagnostic delay, defined as the interval between imaging confirmation of the diagnosis and biopsy; and the time from biopsy to initiation of neoadjuvant systemic therapy. For cases that did not receive neoadjuvant therapy, we calculated the surgical delay, defined as the interval from imaging confirmation or biopsy to surgery. For cases that received neoadjuvant therapy, we measured the post-neoadjuvant interval, defined as the time from completion of systemic therapy to surgery. The interval, expressed in days, between surgery and initiation of adjuvant therapy (chemotherapy, endocrine therapy, or radiotherapy) was defined as the adjuvant delay. For patients receiving chemotherapy, we also calculated the interval between treatment sessions.

Boxplots were generated using available data only; cases with missing values were excluded from the analyses. Zero-day intervals were retained, as they reflect same-day referral and presentation.

### Statistical analysis

Continuous variables, which were non-normally distributed, were described using the median and interquartile range (IQR). Categorical variables were described as percentages. We conducted a comparative analysis between the pandemic and post-pandemic cohorts, using the Mann–Whitney U test for continuous variables and the Chi-square test for categorical variables. A *P* value < 0.05 was considered statistically significant.

Statistical analysis was performed using the Statistical Package for the Social Sciences (SPSS) version 26.0 (IBM Corp., Armonk, NY), and Python-based statistical libraries were used to validate additional results. Cases with incomplete data (due to treatment or imaging performed outside the institute) were excluded from the relevant interval analyses.

## Results

The pandemic cohort included 298 hospitalized breast cancer patients, of whom 73 met the inclusion criteria. The post-pandemic cohort included 343 hospitalized breast cancer patients, of whom 99 met the inclusion criteria. Patient selection for the pandemic and post-pandemic cohorts is shown in Figure 1.

**Figure 1:**
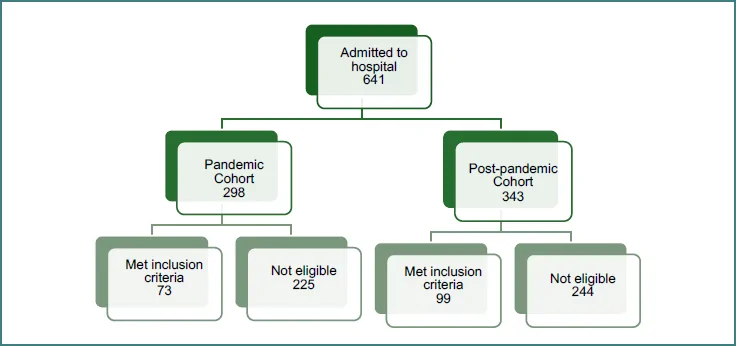
Flowchart of patient selection for the pandemic and post-pandemic cohorts

### Key characteristics of the pandemic cohort (*n* = 73)

For 32 of the 73 patients in the pandemic cohort, the dates of diagnostic breast imaging (ultrasound, mammography, or MRI) were unavailable because the examinations were performed at outside institutions. Within the pandemic cohort, 16 patients underwent surgery without a prior core biopsy, with the diagnosis established intraoperatively by extemporaneous histopathological evaluation. Thirteen patients had undergone biopsy at other healthcare institutions, for which the biopsy dates were unavailable. Neoadjuvant chemotherapy was administered in 43 patients; among these, 10/43 (23.3%) initiated treatment at outside institutions, with incomplete data on treatment initiation and number of cycles. A total of 26 patients received adjuvant chemotherapy, and 20 received postoperative radiotherapy.

### Key characteristics of the post-pandemic cohort (*n* = 99)

For 38 of the 99 patients in the post-pandemic cohort, the dates of diagnostic breast imaging (ultrasound, mammography, or MRI) were unavailable because the examinations had been conducted at outside institutions. In the post-pandemic cohort, 24 patients proceeded to surgery without a prior biopsy, with histopathological confirmation obtained intraoperatively. Twelve patients had undergone biopsy at other healthcare institutions, but no documentation of the biopsy dates was available. Neoadjuvant chemotherapy was administered in 49 patients: among these, 15/49 (30.6%) initiated treatment at outside institutions, with incomplete data on treatment initiation and number of cycles. Adjuvant chemotherapy was administered to 33 patients, while 22 underwent postoperative radiotherapy.

A comparative analysis of characteristics between the pandemic and post-pandemic cohorts is presented in Table 1.

**Table 1:** Comparison of clinical management characteristics between the pandemic and post-pandemic cohorts

Characteristic	Pandemic cohort (*n* = 73)	Post-pandemic cohort (*n* = 99)	*P* value^a^
Diagnostic imaging dates unavailable	32 (43.8)	38 (38.4)	0.574
Direct surgery without prior biopsy	16 (21.9)	24 (24.2)	0.862
Biopsy performed at other institutions	13 (17.8)	12 (12.1)	0.408
Neoadjuvant chemotherapy administered	43 (58.9)	49 (49.5)	0.285
Neoadjuvant therapy initiated at outside institutions^b^	10 (23.3)	15 (30.6)	0.578
Adjuvant chemotherapy administered	26 (35.6)	33 (33.3)	0.881
Postoperative radiotherapy administered	20 (27.4)	22 (22.2)	0.548

a Categorical variables were compared using the χ^2^ test. ^b^ Percentages were calculated relative to the subgroup of patients who received neoadjuvant treatment in each cohort (pandemic, *n* = 43; post-pandemic, *n* = 49).

The baseline demographic and tumor characteristics of the two cohorts are presented in Table 2.

**Table 2:** Baseline demographic and tumor characteristics of the pandemic and post-pandemic cohorts

Variable	Pandemic cohort (*n* = 73)	Post-pandemic cohort (*n* = 99)	*P* value
Age, median [IQR], years	56 [47–67]	59 [50–68]	0.399^a^
Tumor laterality – left, *n* (%)	38 (52.1)	56 (56.6)	0.557
Tumor laterality – right, *n* (%)	35 (47.9)	43 (43.4)	
Tumor location – upper outer quadrant, *n* (%)	34 (46.6)	38 (38.4)	0.449
Tumor location – multicentric/multiple quadrants, *n* (%)	18 (24.7)	24 (24.2)	
Tumor location – other locations, *n* (%)	21 (28.8)	37 (37.4)	
Stage at diagnosis – early stage (0–II), *n* (%)	58 (79.5)	68 (68.7)	0.115
Stage at diagnosis – advanced stage (III–IV), *n* (%)	15 (20.5)	31 (31.3)	

a Compared using the Mann–Whitney U test because of non-normal distribution

The median age was comparable between the pandemic and post-pandemic groups (56 [47–67] vs. 59 [50–68] years, respectively), and median age was similar between groups (*P* = 0.399).

Tumor laterality was similarly distributed across cohorts, with left-sided tumors accounting for 52.1% of cases during the pandemic and 56.6% in the post-pandemic period, while right-sided tumors accounted for 47.9% and 43.4%, respectively.

Regarding tumor location, the upper outer quadrant was the most frequent site in both cohorts (46.6% during the pandemic and 38.4% post-pandemic), followed by multicentric disease (24.7% and 24.2%, respectively). Tumors located in other quadrants (central, upper inner, lower outer, and lower inner) represented 28.8% of cases in the pandemic cohort and 37.4% in the post-pandemic cohort. No statistically significant differences in tumor laterality or location were observed between the two periods.

During the pandemic, 58 of 73 patients (79.5%) were diagnosed with early-stage breast cancer (stages 0–II), while 15 patients (20.5%) presented with advanced-stage disease (stages III–IV). In contrast, in the post-pandemic cohort, 68 of 99 patients (68.7%) were diagnosed at an early stage, whereas 31 patients (31.3%) had advanced-stage disease, as shown in Table 2. Although a higher proportion of advanced-stage cases was observed in the post-pandemic period compared with the pandemic period, this difference did not reach statistical significance (*P* = 0.115). Time intervals across the diagnostic and treatment pathways are summarized in Figures 2 and 3. The interval from referral to first hospital presentation was shorter in the post-pandemic period compared with the pandemic period (3 [1–8] vs. 6 [2–10] days). However, this difference did not reach statistical significance (*P* = 0.053). Imaging-to-biopsy intervals were also reduced post-pandemic (7 [3–22] days, *n* = 43, vs. 14 [4–33] days, *n* = 26, in the pandemic cohort), although this difference did not reach statistical significance (*P* = 0.058). Figure 2AB presents boxplots of diagnostic time intervals (from referral to first presentation and from imaging to biopsy) in the pandemic and post-pandemic cohorts.

**Figure 2:**
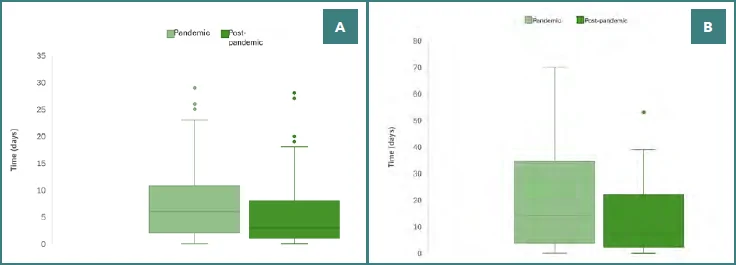
Diagnostic time intervals in the pandemic and post-pandemic cohorts. A, Interval from referral to first hospital presentation. B, Imaging-to-biopsy interval.

As shown in Figure 3A–D, no meaningful differences were observed in the time from biopsy to initiation of neoadjuvant therapy (*P* = 0.651) or from surgery to adjuvant chemotherapy (*P* = 0.942) between the two periods. In contrast, the interval from the end of neoadjuvant therapy to surgery was longer in the post-pandemic cohort (38 [27–49] vs. 33 [28–44] days), although this difference was not statistically significant (*P* = 0.437). Chemotherapy delivery was more efficient post-pandemic, as reflected by significantly shorter intervals between chemotherapy cycles (18 [17–19] vs. 21 [21–22] days; *P* < 0.001). The imaging-to-surgery interval was longer in the post-pandemic cohort (59 [27–80] vs. 28 [20–41] days); however, this difference did not reach statistical significance (*P* = 0.220).

**Figure 3:**
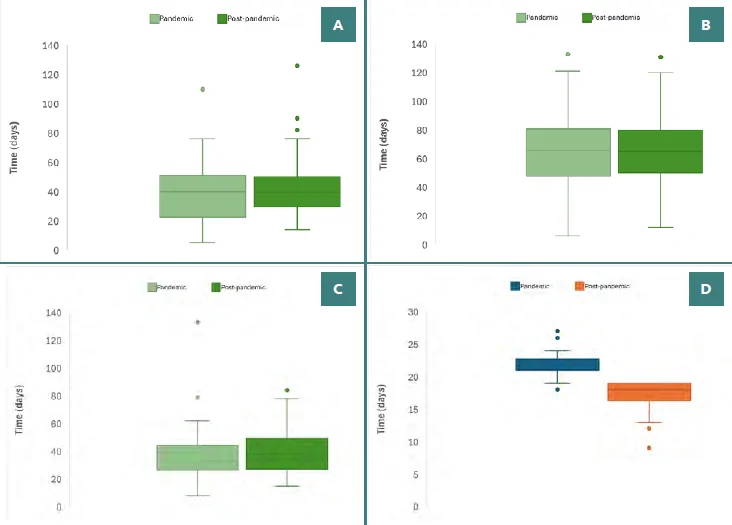
Treatment-related time intervals in the pandemic and post-pandemic cohorts. A, Biopsy to initiation of neoadjuvant therapy. B, Surgery to adjuvant chemotherapy. C, End of neoadjuvant therapy to surgery. D, Interval between chemotherapy cycles.

Tumor grade, receptor status, and molecular subtype distribution are presented in Table 3. No major differences in tumor grade were observed between the two cohorts. Luminal subtypes were predominant in both groups, although a slightly higher proportion of triple-negative and HER2-enriched tumors was observed in the post-pandemic cohort.

**Table 3:** Tumor grade and immunohistochemical characteristics in the pandemic and post-pandemic cohorts

Variable	Pandemic cohort (*n* = 73)	Post-pandemic cohort (*n* = 99)
**Tumor grade**		
G1	6 (8.2%)	14 (14.1%)
G2	50 (68.5%)	61 (61.6%)
G3	17 (23.3%)	24 (24.2%)
**IHC available, n (%)**	40 (54.8%)	77 (77.8%)
ER positive ^a^	34 (85.0%)	58 (75.3%)
PR positive ^a^	33 (82.5%)	54 (70.1%)
HER2 positive ^a^	12 (30.0%)	17 (22.1%)
**Molecular subtype (IHC cases only)^a^**		
Luminal A	17 (42.5%)	29 (37.7%)
Luminal B	18 (45.0%)	34 (44.2%)
HER2-enriched	2 (5.0%)	5 (6.5%)
Triple-negative	3 (7.5%)	9 (11.7%)

Abbreviations: ER, estrogen receptor; HER2, human epidermal growth factor receptor 2; IHC, immunohistochemistry; PR, progesterone receptor. ^a^ Percentages were calculated among patients with available immunohistochemical results only (pandemic, *n* = 40; post-pandemic, *n* = 77).

## Discussion

Breast cancer care at IOB was preserved during the pandemic, despite severe disruptions caused by COVID-19. Treatment timelines, especially initiation of systemic therapy (adjuvant chemotherapy), during the pandemic were comparable to those during the post-pandemic period. This proves that institutional protocols were implemented effectively, including the triage system, neoadjuvant endocrine therapy, and hypofractionated radiotherapy. However, we interpret this with caution, given the selection of surgically eligible patients and the presence of missing immunohistochemical data. International data from the United Kingdom (UK) and the United States (US) reported delays in cancer diagnosis and treatment during the pandemic [12,13]. Similar trends have been reported internationally. Studies from Ireland, Turkey, and Italy described delayed presentation, increased rates of metastatic disease, and more advanced tumor characteristics following pandemic-related disruptions in breast cancer care [14–16]. Overall, our findings are consistent with these observations, although the differences observed in our cohort did not reach statistical significance.

Luminal subtypes, in our pandemic cohort, were predominant, with a low percentage of HER2-enriched and triple-negative tumors. This reflects the selection of patients only eligible for surgical treatment. This distribution suggests that more aggressive or advanced cases—potentially more affected by the pandemic—were underrepresented in our study population. The observed findings may not fully capture the broader impact of the COVID-19 pandemic on breast cancer presentation, particularly among patients with biologically aggressive disease.

Compared with the pandemic cohort, the post-pandemic cohort showed a modest decrease in luminal subtypes and a slight increase in more aggressive phenotypes, including triple-negative and HER2-enriched tumors. Although these differences were not formally tested for statistical significance, they may suggest a shift towards more advanced or biologically aggressive disease in the post-pandemic period.

Several intervals, in our study, appeared shorter in the post-pandemic cohort, including the time from referral to first hospital presentation and from imaging to biopsy. Although our findings did not reach statistical significance, they suggest improved access and efficiency in the diagnostic pathway in the post-pandemic cohort. The wide distribution of imaging-to-biopsy intervals, with several extreme values, suggests heterogeneity in patient pathways, likely related to external investigations.

Treatment-related intervals were comparable between the cohorts, with no significant differences in the time from biopsy to initiation of neoadjuvant therapy or from surgery to initiation of adjuvant chemotherapy. The interval between completion of neoadjuvant therapy and surgery was longer in the post-pandemic cohort, even if this finding was not statistically significant. A longer imaging-to-surgery interval was observed in the post-pandemic cohort; however, this difference did not reach statistical significance, likely due to the limited number of cases.

A significant improvement in chemotherapy delivery was observed, as reflected by shorter intervals between chemotherapy cycles in the post-pandemic period. This may suggest a recovery and possible optimization of the administration of systemic treatment following the constraints imposed by the pandemic.

We did observe a higher percentage of advanced-stage breast cancer cases in the post-pandemic cohort, despite the better chemotherapy schedule and shorter referral to presentation time intervals. However, our findings did not reach statistical significance. Access to primary care was reduced, screening was disrupted, and these changes may have contributed to delayed presentations, which could partly explain the higher proportion of advanced-stage cases reported in previous studies. For example, similar trends were reported in different countries. The Italian COVID-DELAY study demonstrated a rising incidence of late-stage breast cancer after the COVID-19 outbreak, suggesting delayed access to diagnosis and treatment pathways [17]. In Ireland, Higgins *et al*. [16] reported an increase in metastatic presentations and more aggressive tumor characteristics in patients with breast cancer diagnosed after the onset of the pandemic. Studies from Northern Italy also suggested that disruptions in breast cancer screening contributed to delayed diagnosis and stage migration in the post-pandemic period [18]. In England, modeling studies predicted that disruptions in breast cancer screening and diagnostic services would lead to delayed diagnosis, stage migration, and increased breast cancer mortality in the years that follow [19].

Therefore, our findings appear to be consistent with international observations, although our differences did not reach statistical significance. Further studies are needed to evaluate all the causes of advanced-stage cases found in the post-pandemic cohort.

Furthermore, it is important to highlight the role of advanced imaging techniques in complex or atypical breast cancer cases. Although reported only in a small number of patients, cardiac metastases have been described. In these cases, cardiac magnetic resonance (CMR) can provide important diagnostic and prognostic information. Although such cases were not identified in our cohorts, this perspective underscores the evolving role of advanced imaging in breast cancer care during and after the pandemic [20].

### Future research directions

Our findings may help clinicians and hospital administrators improve breast cancer care during future healthcare disruptions. The increase in advanced-stage cases in the post-pandemic cohort we observed suggests the need to maintain access to screening, timely referral pathways, and continuity of surgical and systemic treatment. Future multicenter studies with larger cohorts and long-term follow-up are needed to evaluate survival outcomes, quality of life, and the role of healthcare system recovery strategies in reducing delays and stage migration.

### Strengths and limitations

Our study provides a real-world evaluation of breast cancer care during the COVID-19 pandemic and in the post-pandemic period at a major oncology center in Bucharest, Romania. A direct comparison was performed between two different cohorts that were clearly defined, offering a nuanced view of how breast cancer management was performed under constrained conditions caused by COVID-19.

Our study has several limitations. First, it is a retrospective study, a single-center analysis, and a small patient cohort, so the generalizability of our findings is limited. Second, we have a substantial number of patients who had imaging, biopsy, or even neoadjuvant systemic therapy performed in external facilities. This resulted in incomplete data for some of the intervals. And third, an important limitation of our study is the lack of long-term follow-up. Overall survival, disease-free survival, and quality of life were not assessed. These facts limit the evaluation of the impact that the pandemic had on breast cancer care. Future studies are needed to assess long-term outcomes in this population.

The fact that we excluded the transitional year to achieve a clear separation between the pandemic and post-pandemic period may have led to the omission of an important phase in the recovery of the health care system.

Another key limitation of this study is that it includes only adult female patients with newly diagnosed breast cancer eligible for surgical treatment. This selection may underestimate the full impact that the COVID-19 pandemic had on breast cancer care. An important category of patients (those with delayed presentation, advanced-stage disease, or limited access to healthcare—potentially the most affected by pandemic-related disruptions) is not represented in this cohort.

Our study did not capture psychological distress, economic status, access to primary care, or telemedicine, even though these variables influenced delays in diagnosis and treatment.

## Conclusion

We demonstrated in this study that breast cancer care at IOB remained functional during the COVID-19 pandemic, despite changes in healthcare systems worldwide. Oncology services, neoadjuvant systemic therapy, and adjuvant therapy provided treatment in both periods analyzed, indicating that clinical protocols were effectively adapted. These findings, though, should be interpreted with caution, given the retrospective single-center design of the study, small sample size, the missing data, and lack of statistical significance for some comparisons.

The higher proportion of advanced-stage breast cancer cases in the post-pandemic period highlights the long-lasting impact of the COVID-19 pandemic on health care systems. This likely reflects delays caused by reduced screening and reduced access to primary care. Even though oncology treatment intervals and diagnostic testing improved after the pandemic, surgical timelines were prolonged, especially after neoadjuvant chemotherapy. Our findings underscore the importance of greater surgical capacity and better access to early diagnosis.

## Personal thanks

During the preparation of this manuscript, the authors used Grammarly AI for editing assistance and language improvement, specifically to improve spelling, grammar, word choice, readability, and clarity.
